# Elderly dendritic cells respond to LPS/IFN-γ and CD40L stimulation despite incomplete maturation

**DOI:** 10.1371/journal.pone.0195313

**Published:** 2018-04-13

**Authors:** Joanne K. Gardner, Scott M. J. Cornwall, Arthur W. Musk, John Alvarez, Cyril D. S. Mamotte, Connie Jackaman, Anna K. Nowak, Delia J. Nelson

**Affiliations:** 1 School of Pharmacy and Biomedical Sciences, Curtin University, Bentley, Western Australia (WA), Australia; 2 Curtin Health Innovation Research Institute, Bentley, WA, Australia; 3 Department of Respiratory Medicine, Sir Charles Gairdner Hospital, Nedlands, WA, Australia; 4 The Mount Hospital, Perth, WA, Australia; 5 School of Medicine, University of WA, Nedlands, Perth, WA, Australia; 6 Department of Medical Oncology, Sir Charles Gairdner Hospital, Nedlands, Perth, WA, Australia; Istituto Superiore di Sanità, ITALY

## Abstract

There is evidence that dendritic cells (DCs) undergo age-related changes that modulate their function with their key role being priming antigen-specific effector T cells. This occurs once DCs develop into antigen-presenting cells in response to stimuli/danger signals. However, the effects of aging on DC responses to bacterial lipopolysaccharide (LPS), the pro-inflammatory cytokine interferon (IFN)-γ and CD40 ligand (CD40L) have not yet been systematically evaluated. We examined responses of blood myeloid (m)DC1s, mDC2s, plasmacytoid (p)DCs, and monocyte-derived DCs (MoDCs) from young (21–40 years) and elderly (60–84 years) healthy human volunteers to LPS/IFN-γ or CD40L stimulation. All elderly DC subsets demonstrated comparable up-regulation of co-stimulatory molecules (CD40, CD80 and/or CD86), intracellular pro-inflammatory cytokine levels (IFN-γ, tumour necrosis factor (TNF)-α, IL-6 and/or IL-12), and/or secreted cytokine levels (IFN-α, IFN-γ, TNF-α, and IL-12) to their younger counterparts. Furthermore, elderly-derived LPS/IFN-γ or CD40L-activated MoDCs induced similar or increased levels of CD8^+^ and CD4^+^ T cell proliferation, and similar T cell functional phenotypes, to their younger counterparts. However, elderly LPS/IFN-γ-activated MoDCs were unreliable in their ability to up-regulate chemokine (IL-8 and monocyte chemoattractant protein (MCP)-1) and IL-6 secretion, implying an inability to dependably induce an inflammatory response. A key age-related difference was that, unlike young-derived MoDCs that completely lost their ability to process antigen, elderly-derived MoDCs maintained their antigen processing ability after LPS/IFN-γ maturation, measured using the DQ-ovalbumin assay; this response implies incomplete maturation that may enable elderly DCs to continuously present antigen. These differences may impact on the efficacy of anti-pathogen and anti-tumour immune responses in the elderly.

## Introduction

Aging is associated with alterations in immune function which may contribute to increased susceptibility to infections, cancer and autoimmunity, as well as decreased responses to vaccinations in elderly individuals [1]. Whilst many studies have shown that adaptive immunity declines with age [2, 3, 4], fewer studies have shown that cells of the innate immune system, including dendritic cells (DCs), are also affected by aging [1, 5, 6–9].

Dendritic cells play a key role in initiating and modulating T cell responses [10]. Immature DCs capture antigens and process them for later presentation to T cells. An important function of DCs that enables them to prime/activate T cells is their ability to mature into antigen-presenting cells (APCs) in response to immune stimuli. During an infection, DCs can be stimulated by pathogen-derived molecules (such as lipopolysaccharide; LPS), which act through pattern recognition receptors on DCs (such as Toll-like receptors; TLRs), as well as pro-inflammatory cytokines produced by host cells at an infection site, such as interferon (IFN)-γ. Ligation of the co-stimulatory molecule CD40 on DCs with CD40 ligand (CD40L) on T cells represents another important mechanism of DC activation that is required to “licence” DCs into potent APCs [11]. In the initial stages of maturation/activation, DCs up-regulate antigen-presenting (major histocompatibility complex (MHC)-I and II) and co-stimulatory molecules (such as CD40, CD80 and CD86) and pro-inflammatory cytokines (such as IFN-γ, tumour necrosis factor (TNF)-α, interleukin (IL)-6 and IL-12), which enables them to prime effector T cells [10, 12]. In the later stages DCs and T cells up-regulate inhibitory molecules to facilitate T cell attenuation once an immune response is no longer required [13–15]. Examples of inhibitory DC/T cell interactions include: CD80/CD86 on DCs binding cytotoxic T lymphocyte antigen-4 (CTLA-4) on T cells [16], programmed cell death ligand-1 (PD-L1) on DCs binding programmed cell death protein-1 (PD-1) on T cells [17], lymphocyte activation gene-3 (LAG-3) on T cells binding MHC-II on DCs [18], and galectin-9 (GAL-9) on DCs binding T cell immunoglobulin and mucin-domain containing-3 (TIM-3) on T cells [19]. The adenosine pathway represents another mechanism of suppressive DC/T cell cross-talk [20], as DCs and T cells express the adenosine-producing enzymes CD39 (ectonucleoside triphosphate diphosphohydrolase-1) and CD73 (ecto-5’-nucleotidase), enabling them to generate immunosuppressive adenosine [20]. DCs and T cells can respond to the immunosuppressive effects of adenosine due to expression of the A2A and A2B adenosine receptors [20]. Secretion of anti-inflammatory cytokines, such as IL-10 and transforming growth factor (TGF)-β [15] also mediate suppressive DC/T cell cross-talk. The inhibitory DC/T cell interactions described above lead to suppression of effector T cell cytokine secretion and cytotoxic activity, T cell anergy, and expansion of regulatory T cells (Tregs), thereby limiting effector T cell responses [13, 14, 17], and contributing to the maintenance of immune tolerance [15].

The ability of DCs to respond to activation stimuli may be altered during aging. Of the few studies published to-date, most examined elderly DC responses to TLR ligands/pathogen-derived stimuli, and reported conflicting results with elderly DC responses being increased, maintained or decreased [5, 7, 8, 21–24]. However, during an infection, DCs are activated by a combination of pathogen-derived molecules, and host-derived pro-inflammatory cytokines, such as IFN-γ. No studies have examined the ability of elderly DCs to mature in response to a combination of pathogen-derived stimuli and IFN-γ. Furthermore, the ability of elderly DCs to respond to activation via CD40, which is important for DCs to gain full APC function [11], as well as representing a promising approach for anti-cancer immunotherapy [25], has not yet been reported. Additionally, published studies examining the effects of aging on DCs focused on changes relating to the effector stage of the immune response by measuring antigen-presenting and co-stimulatory molecules, as well as pro-inflammatory cytokines [5, 7, 8, 21–24]. Until now, little attention had been given to inhibitory molecules involved in the attenuation phase of DC/T cell cross-talk.

In this study, we examined the effects of aging on key DC functions involved in the priming/activation and attenuation phases of DC/T cell interactions, including changes in activation and regulatory markers, pro- and anti-inflammatory cytokine production, antigen processing capacity, and an ability to induce functional daughter T cells using blood DC subsets and in vitro monocyte-derived DCs (MoDCs) from young (aged 21–40 years) and elderly volunteers (aged 60–84 years) responding to stimulation with LPS/IFN-γ or CD40L.

## Materials and methods

### Human ethics and participant recruitment

Healthy young (21–40 years of age) and elderly (60–84 years of age) volunteers were recruited by: (i) radio and newspaper advertising, and (ii) poster advertising and word of mouth within Curtin University and rotary clubs. All volunteers gave written informed consent prior to study participation and their health status was assessed via a questionnaire detailing current and past medical conditions, current medications, family disease history, smoking status and asbestos exposure status. Volunteers were excluded from the study if they currently had cancer, autoimmune or other immune disorders, or were taking corticosteroid or immunosuppressive medications. This study was approved by the Human Ethics Committees from Sir Charles Gairdner Hospital, Perth, Western Australia (#2008–041); the Mount Hospital, Perth, Western Australia (#EC50.1), Curtin University, Bentley, Western Australia (#HR68/2008 and #HR102_2012), and the Australian Red Cross Blood Service (#13-03WA-19).

### Blood collection and isolation of peripheral blood mononuclear cells (PBMCs)

Whole blood (50 ml) was collected from healthy young and elderly volunteers into EDTA vacutainers (Becton Dickinson (BD), USA). Three buffy coat samples from healthy male donors aged 23–31 years were collected by the Australian Red Cross Blood Service. The following steps were all performed at room temperature. Whole blood and buffy coats were diluted 1:2 in PBS containing 2 mM EDTA (PBS/EDTA; Sigma-Aldrich, Australia), followed by density gradient centrifugation on Ficoll-Paque^TM^ Plus (GE Healthcare, Australia) at 400 g for 40 minutes. The PBMC layer was collected and washed three times in PBS/EDTA; once at 300 g for 10 minutes, then twice at 200 g for 10 minutes. PBMCs were resuspended in complete medium, consisting of RPMI 1640 media (Thermofisher Scientific, USA) supplemented with 10% fetal calf serum (FCS; Hyclone, Australia), 100 units/ml of penicillin and 100 μg/ml streptomycin (Penicillin-Streptomycin; Thermofisher Scientific, Australia), 2 mM L-glutamax (Thermofisher Scientific), and 0.05 mM 2-mercaptoethanol (Sigma-Aldrich), and aliquoted for: (i) blood DC studies, and (ii) generation of monocyte-derived DCs (MoDCs).

### Blood DC studies

Young and elderly PBMCs (which includes myeloid (m)DC1, mDC2 and plasmacytoid (p)DC subsets) were cultured at 37°C in 96-well plates (BD), at 200 μl per well, in: (i) complete medium only (unstimulated control), or (ii) complete medium supplemented with DC activation stimuli: 1 μg/ml LPS (Sigma-Aldrich) and 20 ng/ml recombinant human IFN-γ (Biolegend, USA) and blood DC subset phenotypes analysed by flow cytometry 24 hours later.

### In vitro generation of MoDCs and lymphocyte isolation

To collect monocytes, PBMCs were incubated in tissue culture flasks or 6-well plates (BD) for 2 hours at 37°C, then non-adherent lymphocytes removed for later use as responder cells in the allogeneic mixed lymphocyte reaction. Adherent monocytes were differentiated into immature MoDCs by culturing for 7 days in complete medium supplemented with 80 ng/ml human granulocyte-macrophage colony-stimulating factor (GM-CSF; Shenandoah Biotechnology, USA), 10 ng/ml recombinant human IL-4 (Shenandoah Biotechnology) and 10 μg/ml Polymixin B (Sigma-Aldrich; to neutralise any LPS potentially present, thereby preventing DC activation during the differentiation period), added on days 0, 4 and 7. On day 7, immature MoDCs were: (i) supplemented with 10 μg/ml Polymixin B (immature/unstimulated control); (ii) stimulated with 1 μg/ml LPS and 20 ng/ml IFN-γ; or (iii) stimulated with 0.66 μg/ml CD40L (Genscript, USA) for a further 2 days. On day 9: (i) MoDC phenotype was analysed via flow cytometry; (ii) MoDC antigen processing capacity was measured using the DQ-ovalbumin (DQ-OVA) assay; (iii) MoDCs were co-cultured with allogeneic T cells in a mixed lymphocyte reaction; and (iv) supernatants from MoDC cultures were collected and stored at -20°C for cytokine bead array analysis.

### DQ-OVA antigen processing assay

MoDCs (5 x 10^4^ cells) in 100 μl of media were incubated with 10 μg/ml DQ-OVA (Thermofisher Scientific) for 1 hour at 4°C or 37°C. Controls consisting of MoDCs without DQ-OVA were included. MoDCs were washed with FACS buffer (PBS containing 1% newborn calf serum (NCS; ThermoScientific) and 1% bovine serum albumin (BSA; Sigma-Aldrich)) and percentages of DQ-OVA^+^ MoDCs analysed by flow cytometry; DQ-OVA is conjugated to BODIPY FL dye and emits fluorescence maximally between 505nm and 515nm.

### Allogeneic mixed lymphocyte reaction

Lymphocytes (isolated from PBMC fractions) were labelled with carboxyfluorescein diacetate succinimidyl ester (CFSE; Thermofisher Scientific), a fluorescent dye that covalently binds to amine groups on molecules in the cytoplasm [26]. Lymphocytes (2 x 10^7^ cells/ml) were incubated in RPMI 1640 media containing 2.5 μM CFSE and no FCS, at room temperature for 10 minutes in the dark and washed three times in complete medium with an FCS underlay by centrifuging at 1,200 rpm for 5 minutes. Young and elderly MoDCs were co-cultured with CFSE-labelled allogeneic young T cells at MoDC: T cell ratios of 1:5, 1:20 and 1:50 for 5–8 days in complete medium at 37°C. Young T cells cultured with 1 μg/ml Concanavalin A (Sigma-Aldrich), a lectin which triggers T cell activation by cross-linking the T cell receptor [27], were used as positive controls. Co-cultures were stained with fluorescently labelled antibodies to identify CD3^+^CD8^+^ and CD3^+^CD4^+^ T cells, and assess T cell functional phenotypes by flow cytometry. As T cells proliferate, CFSE segregates equally between each daughter population; in flow cytometric analysis each round of proliferation is seen as sequential halving of CFSE staining intensity [28]. The percent of T cell proliferation was calculated based on the loss of staining intensity of the parent peak.

### Staining DCs and T cells for phenotyping via flow cytometry

PBMC samples were stained with fluorescently labelled antibodies to identify blood DC subsets (lineage cocktail, CD1c, CD123, CD141, and CD303; Table 1), activation markers (MHC-I, CD40, CD80, CD86, IFN-γ, IL-6, IL-12, and TNF-α; Table 1) and regulatory markers (CD39, CD73, adenosine A2A receptor (A2AR), adenosine A2B receptor (A2BR), PD-L1, GAL-9, IL-10, and TGF-β latency-associated peptide; Table 1).

**Table 1:** Anti-human antibodies used in this study.

**Surface antigens/markers**			
**Antigen**	**Conjugate**	**Clone**	**Supplier**
Adenosine A2B receptor	Purified	Rabbit polyclonal	Alomone Labs (Israel)
CD1a	PE-Cy7	HI149	Biolegend
CD1c	AF647	L161	Biolegend
CD1c	APC-Cy7	L161	Biolegend
CD3	APC-eFluor780	UCHT1	eBioscience (USA)
CD4	BV510	OKT4	Biolegend
CD4	PerCP-Cy5.5	OKT4	Biolegend
CD8	AF647	HIT8a	Biolegend
CD8	PE-Cy7	HIT8a	Biolegend
CD11c	APC	3.9	Biolegend
CD11c	PerCP-Cy5.5	3.9	Biolegend
CD14	FITC	M5E2	BD
CD25	PE-Cy7	BC96	Biolegend
CD39	BV510	A1	Biolegend
CD40	PE	5C3	Biolegend
CD73	BV421	AD2	Biolegend
CD73	PE-Cy7	AD2	Biolegend
CD80	Biotin	2D10	Biolegend
CD80	BV421	2D10	Biolegend
CD86	BV510	IT2.2	Biolegend
CD123	PerCP-Cy5.5	6H6	Biolegend
CD141	BV421	M80	Biolegend
CD303	PerCP-Cy5.5	201A	Biolegend
Lineage cocktail (CD3, CD14, CD16, CD19, CD20, CD56)	FITC	UCHT1, HCD14, 3G8, HIB19, 2H7, HCD56	Biolegend
MHC class I (HLA-A,B,C)	APC-Cy7	W6/32	Biolegend
MHC class II (HLA-DR)	APC-Cy7	L242	BD
PD-L1 (CD274, B7-H1)	PE-Cy7	29E.2A3	Biolegend
**Intracellular antigens/markers**			
**Antigen**	**Conjugate**	**Clone**	**Supplier**
A2A receptor	PE	7F6-G5-A2	Santa Cruz (USA)
A2A receptor	PerCP-Cy5.5	7F6-G5-A2	Santa Cruz
Galectin-9	PE	9M1-3	Biolegend
IFN-γ	BV510	4S.B3	Biolegend
IL-6	PE	MQ2-13A5	Biolegend
IL-10	BV421	JES3-97D	Biolegend
IL-10	PE-Cy7	JES3-97D	Biolegend
IL-12 (p40/p70)	APC	C11.5	BD
TGF-β1 latency-associated peptide	PE-Cy7	TW4-2F8	Biolegend
TNF-α	APC-Cy7	MAb11	Biolegend

For phenotypic analysis of MoDCs, samples were stained with antibodies to identify MoDCs (CD11c and CD14; Table 1), activation markers (MHC-I, MHC-II, CD1a, CD40, CD80, CD86, IFN-γ, IL-6, IL-12, and TNF-α; Table 1) and regulatory markers (CD39, CD73, A2AR, A2BR, PD-L1, GAL-9, IL-10, and TGF-β latency-associated peptide; Table 1).

CD8^+^ and CD4^+^ T cells from MLR co-cultures were stained with antibodies to assess markers of activation/effector (CD25 and IL-12; Table 1) and regulatory function (A2AR, CD39, CD73, and intracellular IL-10 and TGF-β latency-associated peptide; Table 1).

For samples requiring intracellular cytokine staining, cells were incubated with 10 μg/ml brefeldin A (Sigma-Aldrich) for 4 hours at 37°C prior to staining, to inhibit protein/cytokine secretion [29]. Single cell suspensions were incubated with surface primary antibodies (diluted in FACS buffer + 2.5 μg/ml brefeldin A) for 30 minutes at 4°C in the dark, and washed twice in FACS buffer + 2.5 μg/ml brefeldin A by centrifuging at 1,200 rpm for 2 minutes. Samples stained with purified A2BR antibody or CD80-biotin were incubated with goat anti-rabbit IgG- AlexaFluor® 488 (Thermofisher Scientific) or streptavidin-BD Horizon™ V500 (BD), respectively (diluted in FACS buffer + 2.5 μg/ml brefeldin A), for 30 minutes at 4°C in the dark, washed twice in FACS buffer + 2.5 μg/ml brefeldin A, followed by two PBS washes. Cells were incubated with Zombie Green or Zombie NIR™ fixable viability dyes (both from Biolegend) for 15 minutes at 4°C in the dark, washed twice in FACS buffer, then twice in PBS. Cells were fixed in 1% paraformaldehyde (Sigma) diluted in PBS by incubating for 20 minutes at 4°C in the dark, washed twice with FACS buffer, permeabilised with FACS buffer + 0.1% saponin (Sigma) for 15 minutes at 4°C in the dark, stained with intracellular antibodies (diluted in FACS buffer + 0.1% saponin) for 30 minutes at 4°C in the dark, washed twice in FACS buffer and resuspended in 200 μl of FACS buffer per well. Samples were analysed immediately, or stored overnight at 4°C in the dark (up to a maximum of 1 week) before acquisition on a FACSCanto II followed by analysis using FACSDiva software (BD) or FlowJo software (TreeStar, USA).

### Cytokine bead array (CBA)

MoDC culture supernatants were tested for TNF-α, IL-10, IL-12p70, VEGF and IFN-γ using a CBA kit from BD, as per the manufacturer’s protocol. Concentrations of IFN-α, IFN-γ, TNF-α, IL-1β, IL-6, IL-8, IL-10, IL-12p70, IL-17A, IL-18, IL-23, IL-33, and monocyte chemoattractant protein-1 (MCP-1) were measured using a LEGENDplex Human Inflammation Panel CBA (Biolegend). The LEGENDplex CBA was optimised so that each test could be performed using half of the manufacturer’s recommended volumes for samples and reagents, and all other aspects of the assay were performed as per the manufacturer’s instructions. Samples were acquired on a FACSCanto II using FACSDiva software, and analysed using FlowJo software or LEGENDplex software (Biolegend).

### Statistics

Statistical differences were calculated using: (i) a Mann-Whitney *U*-test to compare young and elderly populations; or (ii) a paired *t*-test to compare observations within each volunteer, using GraphPad PRISM v7 (GraphPad Software Inc, USA). *P*-values less than 0.05 were considered statistically significant.

## Results

### Elderly MoDCs have increased CD1a, CD40 and CD86 after CD40 stimulation

MoDCs that differentiate from monocytes at sites of inflammation can promote effector T cell responses. In this scenario, inflammation may drive CD40L upregulation on CD4^+^ T cells leading to CD40L-CD40 interactions with DCs, rendering DCs more effective antigen presenting cells. Activating DCs through CD40 also represents a promising anti-cancer immunotherapeutic approach. Therefore, we investigated whether elderly MoDCs respond to CD40 activation signals. Monocytes from young (21–40 years of age) and elderly (60–84 years of age) healthy volunteers differentiated into immature MoDCs were left unstimulated or stimulated with CD40L, and analysed using flow cytometry, as per S1A–S1E Fig. No age-related differences in percentages of viable immature/unstimulated or CD40L-stimulated CD11c^+^CD14^-^ MoDCs were observed (data not shown).

**Fig 1:**
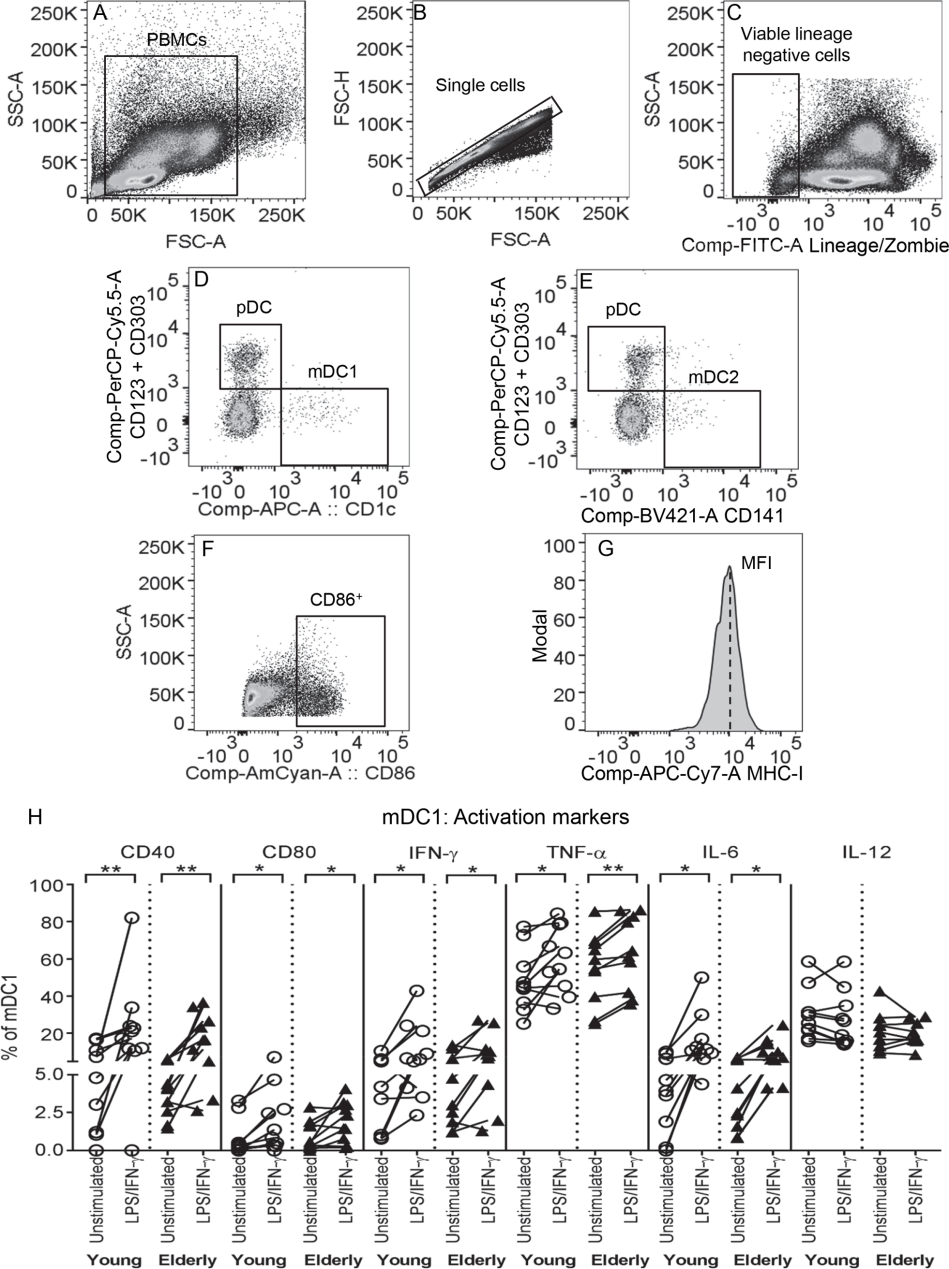
Young and elderly mDC1s increase CD40, CD80, IFN-γ, TNF-α and IL-6 after LPS/IFN-γ. Young and elderly PBMCs were left unstimulated or stimulated with LPS/IFN-γ for 24 hours, then stained with a lineage cocktail (containing CD3, CD14, CD16, CD19, CD20 and CD56), markers of blood DC subsets (CD1c, CD141, CD123 and CD303), and activation markers (CD40, CD80, and intracellular IFN-γ, TNF-α, IL-6 and IL-12) for flow cytometric analysis. Within PBMC (A), single cells (B) and viable lineage negative cells (C) gates, blood DC subsets were gated as: mDC1s (CD1c^+^CD123^-^CD303^-^; D), mDC2s (CD141^+^CD123^-^CD303^-^; E) and pDCs (CD123^+^CD303^+^CD1c^-^; D or CD123^+^CD303^+^CD141^-^; E). Marker expression on blood DCs was measured using percentage of cells positive (representative graph shown in F) and geometric mean fluorescence intensity (MFI) expression levels (representative graph shown in G). In graph (H), each line represents an individual volunteer, and compares the percentage of mDC1s positive for each activation marker in their LPS/IFN-γ-stimulated sample to their unstimulated control. Statistical comparisons were also performed between young and elderly volunteers within each condition. Data shown as individual values, n = 10 young volunteers, n = 10 elderly volunteers, * = p<0.05, ** = p<0.005, *** = p<0.0005 comparing LPS/IFN-γ-mDC1s to unstimulated mDC1s from the same volunteer.

To assess whether elderly CD40-activated MoDCs were functional, expression of antigen-presenting molecules (MHC-II and CD1a) and co-stimulatory molecules (CD40, CD80 and CD86) were measured, as per S1E Fig. Following CD40L stimulation, percentages of MHC-II^+^ MoDCs were not altered in either age group (S1F Fig). In contrast, increased percentages of elderly MoDCs expressing CD1a (p = 0.006; S1F Fig) and CD40 (p = 0.0003; S1F Fig), relative to each volunteer’s own unstimulated controls were seen. The latter did not occur in young MoDCs (S1F Fig) accounting for the higher percentage of CD40^+^ elderly MoDCs compared to young MoDCs (p = 0.01; S1F Fig). Whilst CD40L-stimulation led to increased percentages of CD80^+^ (p = 0.02, p = 0.004; Fig 1F) and CD86^+^ (p = 0.002, p = 0.001; S1F Fig) MoDCs in both age groups, changes in CD86 were greater for elderly MoDCs resulting in a higher percentage of CD86^+^ elderly MoDCs compared to young MoDCs (p = 0.003; S1F Fig). Expression levels of these markers (measured via geometric mean fluorescence intensity; MFI) showed similar trends (data not shown).

As cytokine secretion is a powerful indicator of DC function, we measured concentrations of pro-inflammatory (IFN-γ, IL-12p70 and TNF-α) and anti-inflammatory cytokines (IL-10 and vascular endothelial growth factor; VEGF) secreted by young and elderly CD40L-stimulated MoDCs to determine whether there were any age-related differences. These cytokines were not detected in culture supernatants of young or elderly immature unstimulated MoDCs (S1G Fig). No age-related differences were seen as both young and elderly CD40L-activated MoDCs up-regulated IFN-γ, TNF-α and VEGF secretion, compared to age-matched unstimulated controls (S1G Fig).

With aging, the generation of effector T cell responses declines and one contributing factor could be age-related changes in the ability of DCs to stimulate T cells. To determine whether elderly MoDCs were defective in stimulating T cells, young (n = 5–7) and elderly (n = 9–11) immature/unstimulated and CD40L-stimulated MoDCs were co-cultured with allogeneic, CFSE-labelled T cells from young volunteers (aged 22–34 years). CD8^+^ and CD4^+^ T cell proliferation was measured using flow cytometry (S2A–S2E Fig), and the percentages of daughter T cells that had undergone at least one round of division/proliferation (S2E Fig) compared. Neither young nor elderly CD40L-activated MoDCs induced significant CD4^+^ and CD8^+^ T cell proliferation (data not shown). Taken together, these data suggest that age does not interfere with the ability of elderly MoDCs to respond to CD40L stimulation, and that inducing significant levels of T cell proliferation requires DCs given a different form of stimulation.

### Elderly mDC1s increase TGF-β and elderly pDCs increase PD-L1 after LPS/IFN-γ stimulation

**Fig 2:**
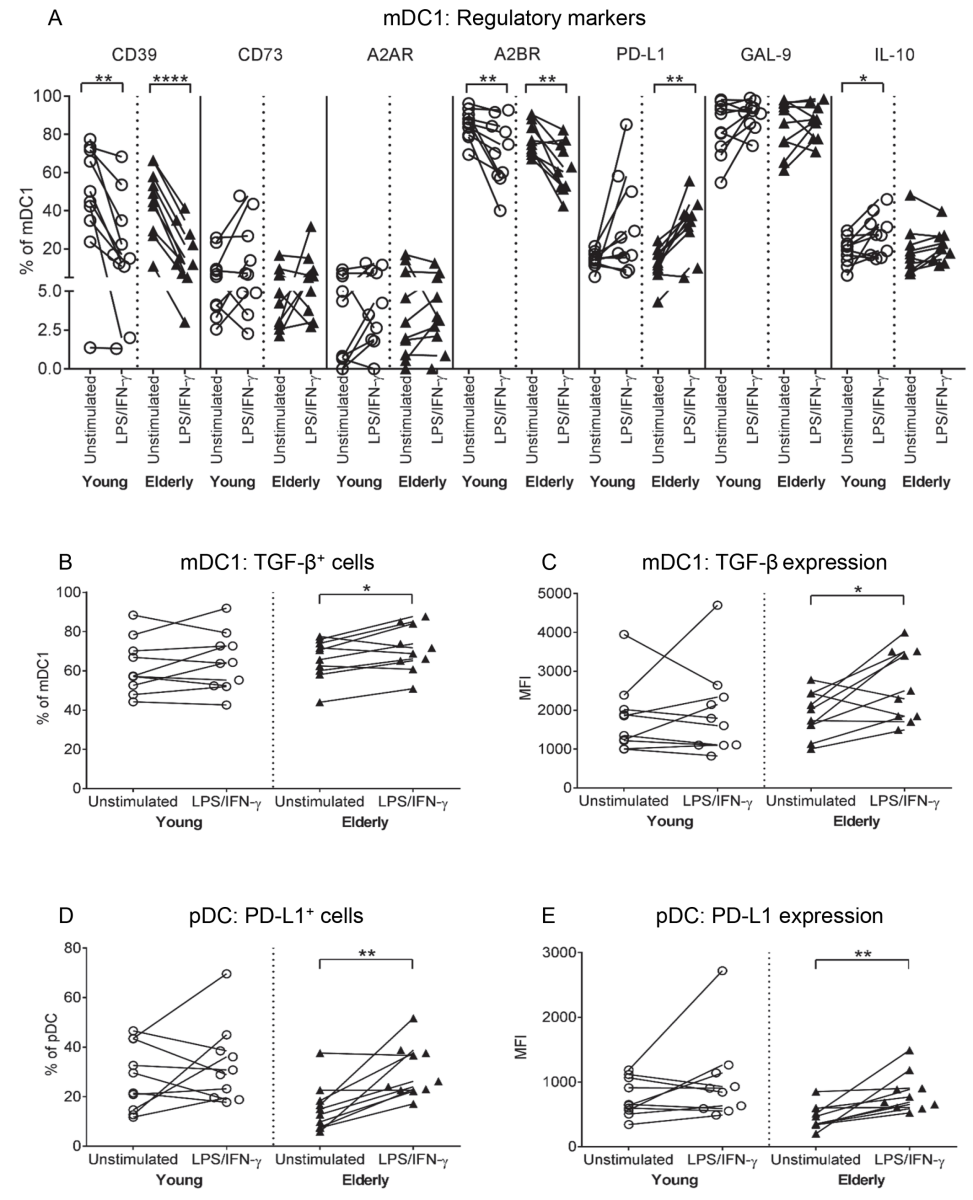
Elderly mDC1s increase TGF-β and elderly pDCs increase PD-L1 after LPS/IFN-γ. Young and elderly PBMCs were left unstimulated or stimulated with LPS/IFN-γ for 24 hours, and analysed via flow cytometry for CD1c^+^ mDC1s and CD123^+^CD303^+^ pDCs, and expression of regulatory markers (CD39, CD73, A2AR, A2BR, PD-L1, GAL-9, and intracellular IL-10 and latent TGF-β), as per Fig 1A–1G. Percentages of mDC1s positive for regulatory markers (A), TGF-β (B) and TGF-β expression levels (C), and percentages of PD-L1^+^ pDCs (D) and PD-L1 expression levels (E) were measured. Each line in (A-E) represents an individual volunteer, and compares their LPS/IFN-γ-stimulated sample to their unstimulated control. Statistical comparisons were also performed between young and elderly volunteers within each condition. Data shown as individual values, n = 10 young volunteers, n = 10 elderly volunteers, * = p<0.05, ** = p<0.005, **** = p<0.0001 comparing LPS/IFN-γ-DCs to unstimulated DCs from the same volunteer.

DCs mature in response to pathogen-derived stimuli, such as LPS, and inflammatory cytokines such as IFN-γ produced by local immune cells, in order to generate an anti-pathogen immune response. We first investigated whether circulating elderly DC subsets, that is mDC1s (which activate T helper (Th)-1 responses), mDC2s (which cross-present antigens and activate effector CD8^+^ T cells, and pDCs (which mediate innate/anti-viral immunity) [30], responded to LPS/IFN-γ stimulation to the same extent as DCs from young volunteers. PBMC suspensions from young and elderly volunteers were left unstimulated, or stimulated with LPS/IFN-γ, before analysis by flow cytometry for expression of molecules associated with antigen presentation and T cell activation (MHC-I, CD40, CD80 and CD86, as well as intracellular IFN-γ, TNF-α, IL-6 and IL-12) and regulation (enzymes and receptors of the adenosine pathway: CD39, CD73, A2AR and A2BR, as well as PD-L1, GAL-9, and intracellular IL-10 and TGF-β latency-associated peptide); representative graphs shown in Fig 1A–1G. We did not observe any age-related differences in the markers examined on unstimulated mDC1s, mDC2s and pDCs (Figs 1H and 2A, S3A and S3B, S4A and S4B).

A comparison of LPS/IFN-γ-stimulated versus unstimulated mDC1s within each volunteer showed that young and elderly mDC1s experienced similar changes in antigen-presenting/activation markers following LPS/IFN-γ stimulation; i.e. percentages of mDC1s expressing MHC-I and CD86 decreased (not shown), whilst percentages of mDC1s positive for CD40, CD80, IFN-γ, TNF-α and IL-6 increased (Fig 1H). Concomitantly, both age groups increased percentages of mDC1s expressing the regulatory marker PD-L1, yet decreased the adenosine-producing enzyme CD39 and the A2B receptor (Fig 2A). Sixty to 70% of elderly mDC1s up-regulated latent TGF-β in terms of percentage of cells positive, as well as expression levels, in response to LPS/IFN-γ stimulation, compared to their unstimulated controls (p = 0.03; Fig 2B; p = 0.03; Fig 2C), with lower numbers (40–50%) of young-derived LPS/IFN-γ-stimulated mDC1s demonstrating a similar increase in TGF-β (Fig 2B and 2C). However, statistical analyses comparing young to elderly mDC1 TGF-β expression within unstimulated and LPS/IFN-γ-stimulated groups did not reach significance, making it difficult to interpret this data. Therefore, it is possible that the age-related differences seen are not physiologically significant, further studies in a larger cohort are required to draw a firm conclusion.

Young and elderly mDC2s demonstrated similar responses to LPS/IFN-γ stimulation by up-regulation of activation (CD80, TNF-α, IL-6, IL-12; S3A Fig) and regulatory (PD-L1 and IL-10; S3B Fig) markers, relative to unstimulated controls.

The only age-related difference in pDC responses to LPS/IFN-γ stimulation was that 80–100% of elderly volunteers up-regulated PD-L1 (p = 0.004; Fig 2D; and p = 0.008; Fig 2E), relative to their own unstimulated controls (Fig 2D and 2E). In contrast, 50% of young-derived pDCs up-regulated PD-L1 following LPS/IFN-γ stimulation (Fig 2D and 2E). However, statistical analyses comparing young to elderly pDC PD-L1 expression within unstimulated and LPS/IFN-γ-stimulated groups did not reach significance, suggesting this change in PD-L1 may not be meaningful in the aging setting. Both young and elderly pDCs demonstrated similar changes in the other activation (S4A Fig) and regulatory markers (S4B Fig), i.e. increased CD40, IL-6 and IL-12 (S4A Fig), and decreased A2BR (S4B Fig).

In summary, elderly blood DC subsets demonstrated comparable up-regulation of co-stimulatory CD40 and CD80, and pro-inflammatory IFN-γ, TNF-α, IL-6 and/or IL-12, to their younger counterparts.

### LPS/IFN-γ-stimulated MoDCs express similar activation and regulatory markers, regardless of age

**Fig 3:**
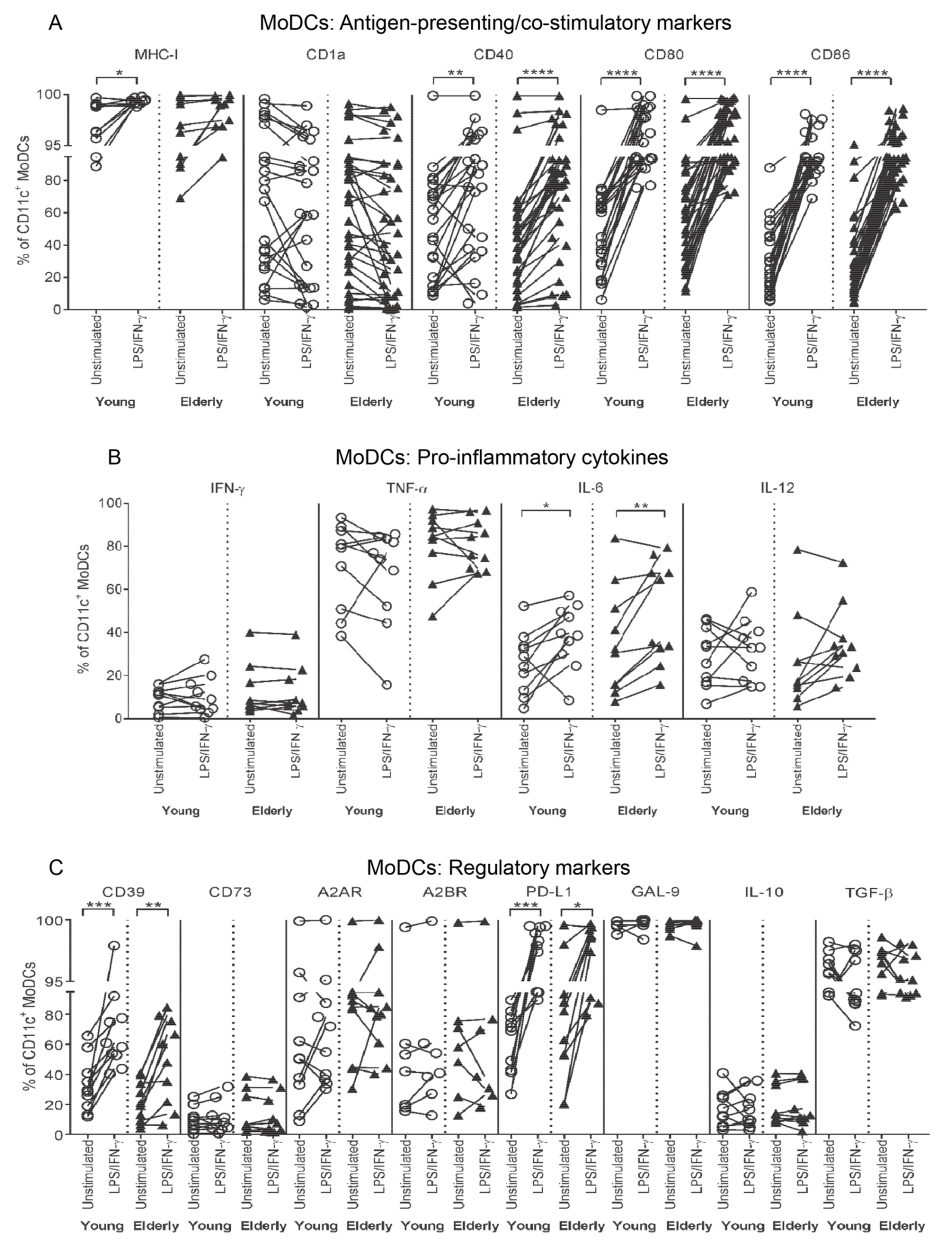
Young and elderly LPS/IFN-γ-MoDCs up-regulate CD40, CD80, CD86, IL-6, CD39 and PD-L1. Young and elderly monocytes differentiated into immature CD11c^+^CD14^-^ MoDCs using GM-CSF and IL-4 for seven days, were left unstimulated or stimulated with LPS/IFN-γ for a further two days, and analysed via flow cytometry for antigen-presenting and co-stimulatory markers (MHC-I, CD1a, CD40, CD80 and CD86), intracellular pro-inflammatory cytokines (IFN-γ, TNF-α, IL-6 and IL-12), and regulatory markers (CD39, CD73, A2AR, A2BR, PD-L1 and GAL-9, and intracellular IL-10 and latent TGF-β). Percentages of CD11c^+^CD14^-^ MoDCs positive for antigen-presenting and co-stimulatory molecules (A), pro-inflammatory cytokines (B), and regulatory markers (C) were measured; each line in A-C represents an individual volunteer, and compares their LPS/IFN-γ-stimulated sample to their unstimulated control. Statistical comparisons were also performed between young and elderly volunteers within each condition. Data shown as individual values, n = 7–23 young volunteers, n = 7–34 elderly volunteers, * = p<0.05, ** = p<0.005, *** = p<0.0005, **** = p<0.0001 comparing LPS/IFN-γ-MoDCs to unstimulated MoDCs from the same volunteer.

We also examined the ability of young and elderly MoDCs to mature in response to LPS/IFN-γ stimulation. Monocytes from young and elderly volunteers were differentiated into immature MoDCs, then stimulated with LPS/IFN-γ, and analysed as per S1A–S1E Fig. Young and elderly immature/unstimulated MoDCs displayed similar expression of activation and regulatory markers (Fig 3A–3C) and responded similarly to LPS/IFN-γ; i.e. percentages of cells positive for MHC-I, CD40, CD80 and CD86, as well as intracellular IL-6, increased relative to their unstimulated controls (Fig 3A and 3B). Percentages of MoDCs positive for regulatory CD39 and PD-L1 also increased after LPS/IFN-γ stimulation, regardless of age (Fig 3C).

### Young but not elderly LPS/IFN-γ-MoDCs consistently secrete MCP-1, IL-6 and IL-8

**Fig 4:**
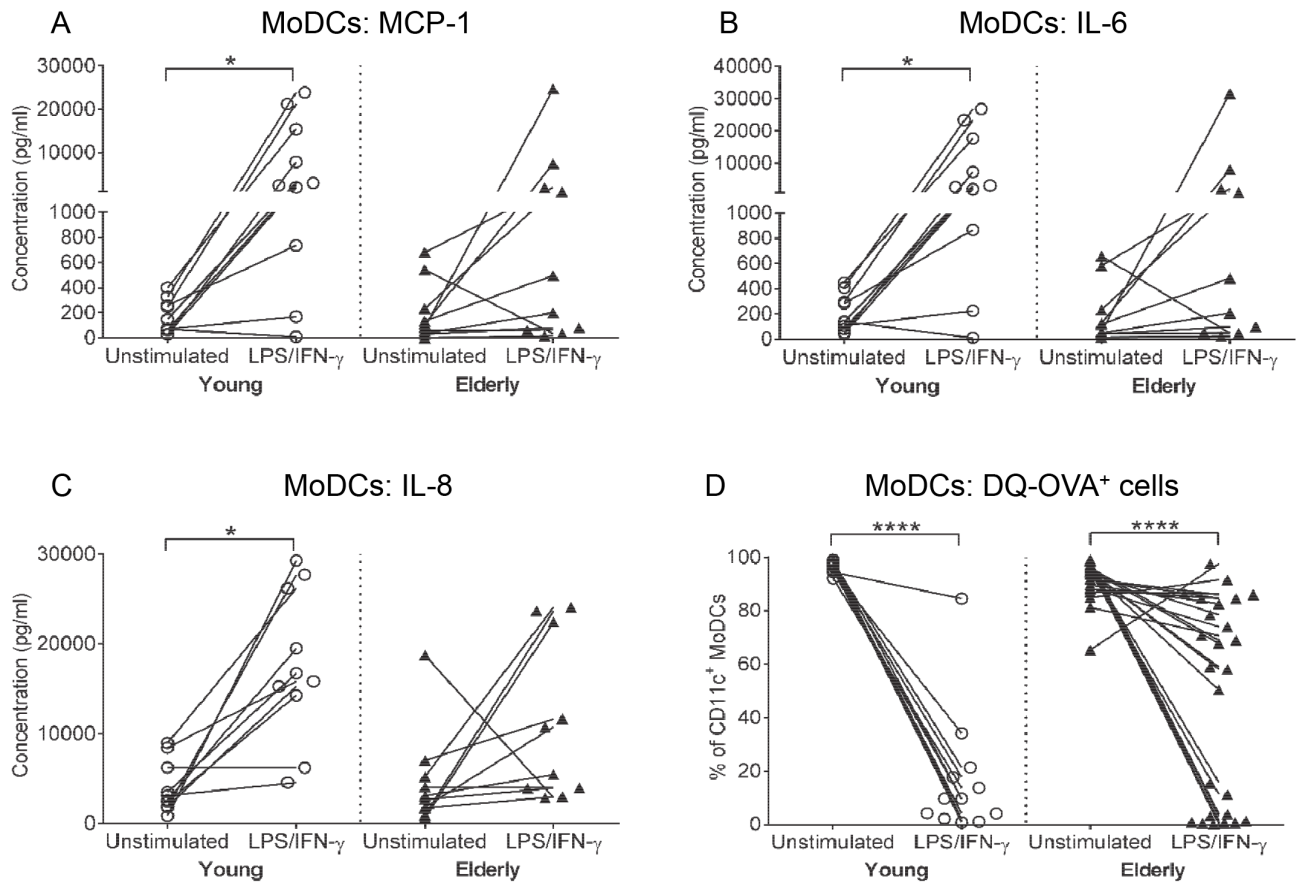
Elderly LPS/IFN-γ-MoDCs have variable changes in MCP-1, IL-6 and IL-8 secretion and partially down-regulate antigen-processing capacity. Concentrations of MCP-1 (A), IL-6 (B), and IL-8 (C) were measured in culture supernatants from unstimulated/immature and LPS/IFN-γ-stimulated young and elderly MoDCs via cytokine bead array. Antigen processing ability of young and elderly MoDCs was assessed by incubating MoDCs with a fluorescent ovalbumin conjugate (DQ-OVA), and measuring the percentage of DQ-OVA^+^ cells via flow cytometry (D). Each line in (A-D) represents an individual volunteer, and compares their LPS/IFN-γ-stimulated sample to their unstimulated control. Statistical comparisons were also performed between young and elderly volunteers within each condition. Data shown as individual values, n = 10–12 young volunteers, n = 10–25 elderly volunteers, * = p<0.05, **** = p<0.0001 comparing LPS/IFN-γ-MoDCs to unstimulated MoDCs from the same volunteer.

Levels of pro-inflammatory cytokines (IFN-α, IFN-γ, TNF-α, IL-1β, IL-6, IL-12p70, IL-17A, IL-18, IL-23 and IL-33), chemokines (MCP-1 and IL-8), and anti-inflammatory cytokines (IL-10 and VEGF) secreted by LPS/IFN-γ-stimulated MoDCs were measured. Immature/unstimulated young and elderly MoDCs secreted similar levels of MCP-1, IL-6 and IL-8 (Fig 4A–4C). Both age groups up-regulated IFN-α, IFN-γ, TNF-α, IL-12p70 and VEGF secretion following LPS/IFN-γ stimulation (S5A and S5B Fig). However, only young LPS/IFN-γ-MoDCs significantly up-regulated secretion of MCP-1 (p = 0.03; Fig 4A), IL-6 (p = 0.03; Fig 4B) and IL-8 (p = 0.001; Fig 4C), compared to their unstimulated controls, whilst inconsistent changes were seen for elderly LPS/IFN-γ-MoDCs (Fig 4A–4C). These data suggest that MoDCs from elderly volunteers are heterogeneous with some individuals displaying a reduced ability to up-regulate pro-inflammatory cytokine/chemokine secretion in response to LPS/IFN-γ stimulation.

### LPS/IFN-γ-activated elderly MoDCs maintain antigen processing capacity

The primary role of immature DCs is to internalize and process antigen, which triggers their maturation. Once mature, DCs lose their ability to process antigen. The antigen processing ability of young and elderly immature/unstimulated and LPS/IFN-γ-stimulated MoDCs was determined using the DQ-OVA assay. This assay measures antigen processing by fluorescence visualized in the FITC channel of a self-quenching ovalbumin conjugate following proteolytic degradation. No significant age-related differences were seen in the percentages of immature/unstimulated MoDCs able to process antigen, as 80–100% of immature MoDCs from both age groups were positive for DQ-OVA (Fig 4D).

Ninety percent of young-derived LPS/IFN-γ-stimulated MoDCs demonstrated an almost complete loss of DQ-OVA expression (i.e. antigen processing ability) compared to their unstimulated controls (p < 0.0001; Fig 4D). In contrast, only 40% of elderly-derived LPS/IFN-γ-stimulated MoDCs showed a similar loss of DQ-OVA expression (Fig 4D), with the majority demonstrating a partial reduction (Fig 4D), suggesting maintenance of their ability to process antigen, and partial maturation. These data imply that whilst elderly MoDCs respond to LPS/IFN-γ, they do not mature and lose antigen processing capacity at equivalent levels to their younger counterparts.

### Elderly LPS/IFN-γ-MoDCs have an increased ability to stimulate T cell proliferation

To generate effective immune responses, appropriately activated mature DCs must be able to stimulate effector T cell proliferation. To determine whether elderly LPS/IFN-γ-activated MoDCs are defective in stimulating T cells, young and elderly LPS/IFN-γ-MoDCs were co-cultured with allogeneic, CFSE-labelled T cells from young volunteers (aged 22–34 years), and CD8^+^ and CD4^+^ T cell proliferation measured using flow cytometry, as per S2A–S2E Fig.

**Fig 5:**
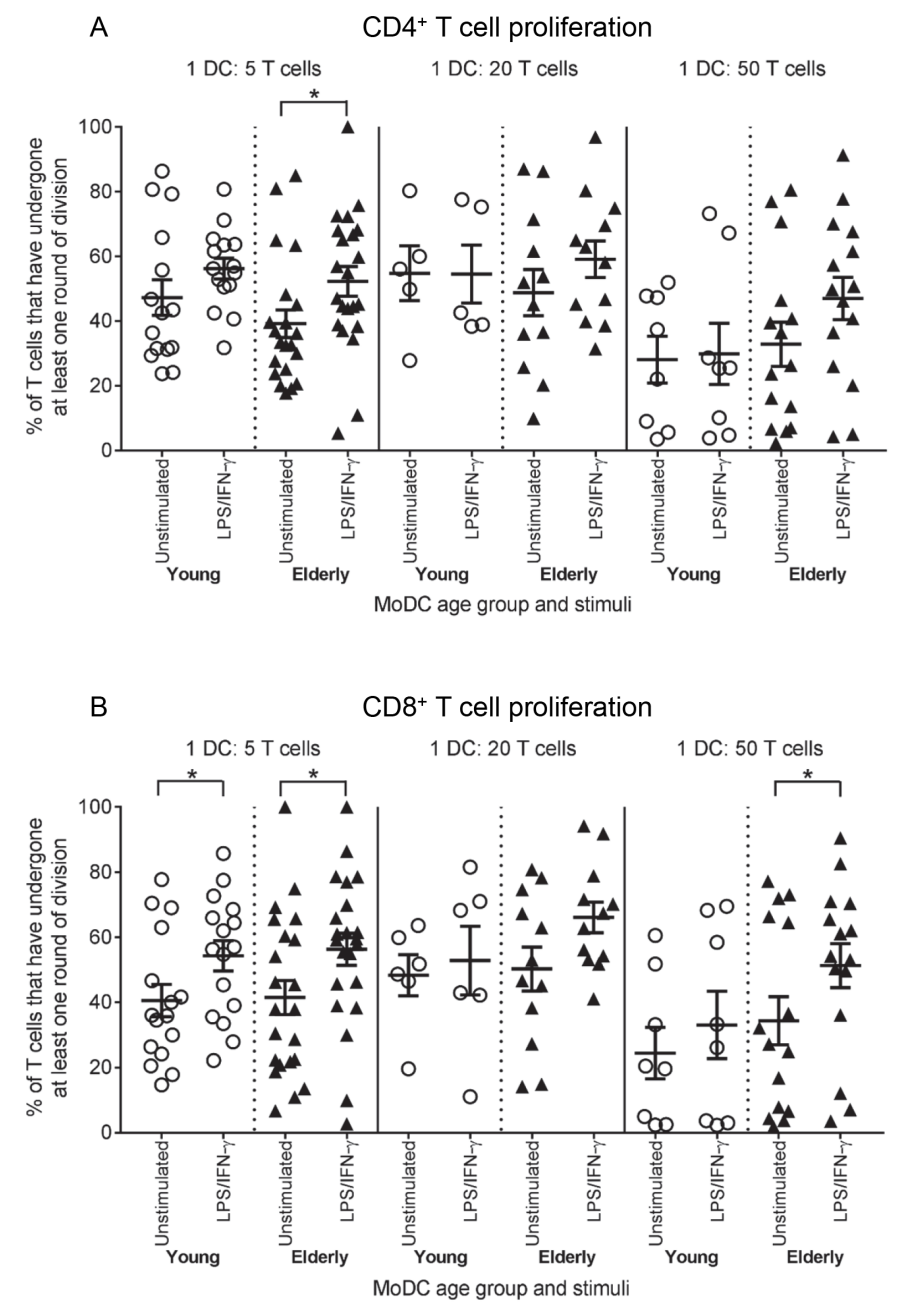
Elderly LPS/IFN-γ-MoDCs induce greater T cell proliferation at lower DC: T cell ratios. Young and elderly immature/unstimulated and LPS/IFN-γ-stimulated MoDCs were co-cultured with allogeneic, CFSE-labelled young T cells at DC: T cell ratios of 1:5, 1:20 and 1:50 for 5–8 days, and T cell proliferation analysed via flow cytometry as per S2A–S2E Fig. Percentages of young CD4^+^ (A) and CD8^+^ T cell (B) proliferation induced by LPS/IFN-γ-MoDCs were compared to those induced by age-matched unstimulated MoDCs. Statistical comparisons were also performed between young and elderly volunteers within each condition. Data shown as individual values, n = 4–16 young volunteers’ MoDCs, n = 9–22 elderly volunteers’ MoDCs, * = p<0.05 comparing LPS/IFN-γ-MoDCs to age-matched unstimulated MoDCs.

Young and elderly immature/unstimulated MoDCs, which are poor stimulators of T cells, induced similar levels of CD4^+^ (Fig 5A) and CD8^+^ T cell (Fig 5B) proliferation at all DC: T cell ratios examined.

At a ratio of 1 DC:5 T cells, both young and elderly LPS/IFN-γ-stimulated MoDCs induced significantly higher levels of CD4^+^ T cell (p = 0.05, p = 0.01, respectively; Fig 5A) and CD8^+^ T cell (p = 0.03, p = 0.01, respectively; Fig 5B) proliferation, compared to age-matched unstimulated MoDCs. Interestingly, elderly LPS/IFN-γ-MoDCs demonstrated a trend for stimulating increased levels of CD4^+^ T cell proliferation at a ratio of 1 DC:50 T cells, relative to their age-matched unstimulated counterparts (p = 0.08; Fig 5A). Additionally, at ratios of 1 DC:20 T cells and 1 DC:50 T cells, elderly LPS/IFN-γ-MoDCs stimulated increased levels of CD8^+^ T cell proliferation, relative to age-matched unstimulated MoDCs; this reached significance at the 1:50 ratio (p = 0.03; Fig 5B). These data suggest that elderly DCs are better equipped to stimulate T cell proliferation at lower DC:T cell ratios.

### Young and elderly LPS/IFN-γ-MoDCs induce T cells with the same phenotype

Measuring T cell proliferation does not reveal the functional quality of activated T cells. To determine whether young CD8^+^ and CD4^+^ T cells stimulated by elderly LPS/IFN-γ-MoDCs were functional, T cells from co-cultures of 1 MoDC:5 T cells were stained for markers of effector function/activation (CD25 and intracellular IFN-γ, IL-12 and perforin), and regulatory markers (CD39, CD73, A2AR, CTLA-4, inducible T cell co-stimulator (ICOS), TIM-3, PD-1, and intracellular IL-10 and TGF-β latency-associated peptide), and analysed using flow cytometry. Changes in marker expression from parent to daughter T cell populations, induced by young versus elderly LPS/IFN-γ-MoDCs, were compared.

**Fig 6:**
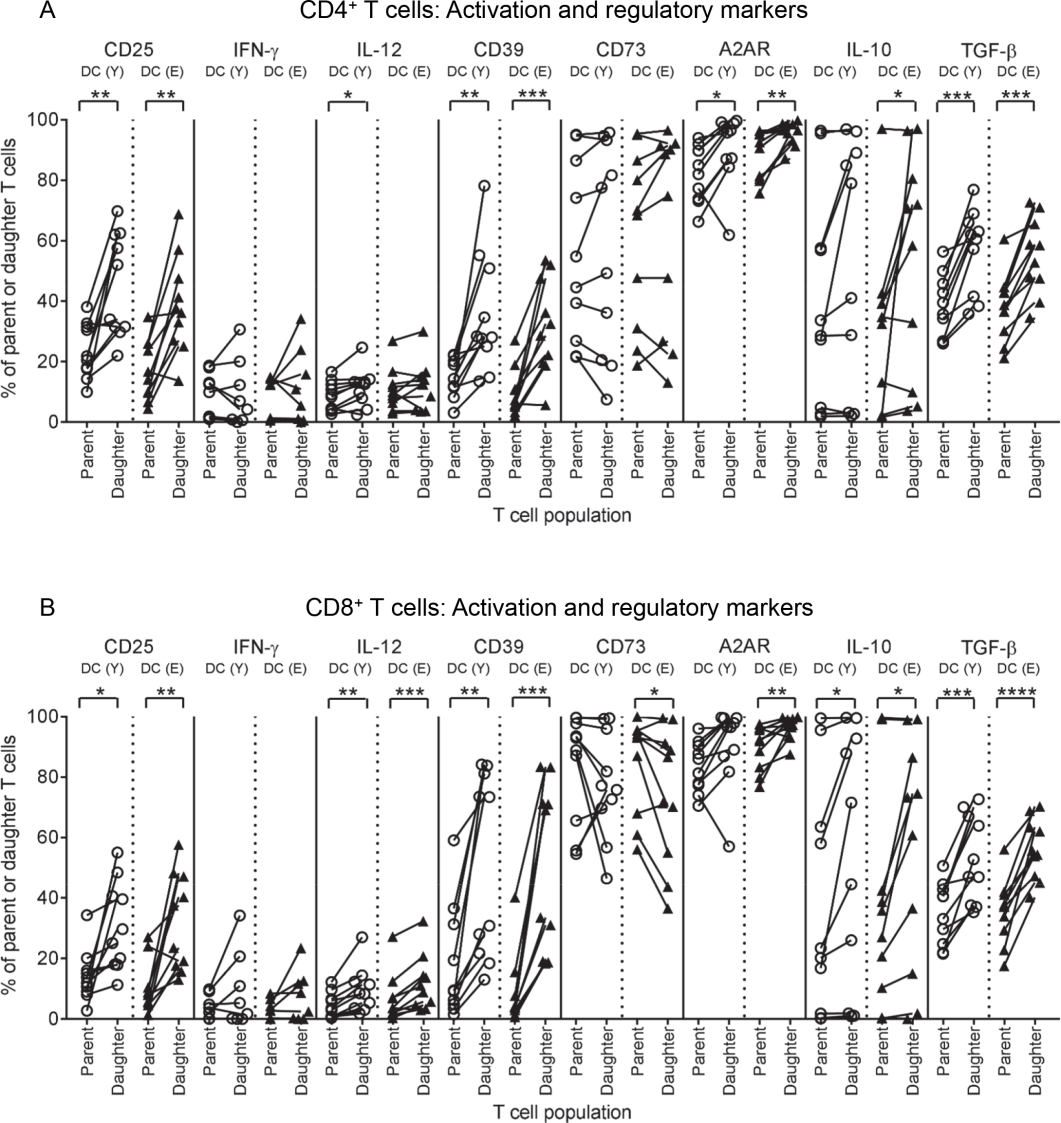
Young and elderly LPS/IFN-γ-MoDCs induce up-regulation of CD25, IL-12, CD39, A2AR, IL-10 and TGF-β on daughter T cells. Young CD4^+^ and CD8^+^ T cells were stimulated by young and elderly LPS/IFN-γ-MoDCs in an MLR assay, at a ratio of 1 MoDC: 5 T cells, as described in Fig 5. Percentages of parent and daughter CD4^+^ (A) and CD8^+^ T cells (B) positive for activation markers (CD25 and intracellular IFN-γ and IL-12), and regulatory markers (CD39, CD73, A2AR, and intracellular IL-10 and latent TGF-β) were measured using flow cytometry. Changes in marker expression from parent to daughter CD4^+^ (A) and CD8^+^ T cells (B) were compared for samples stimulated by young versus elderly LPS/IFN-γ-MoDCs, where each line represents the change in marker expression from parent to daughter T cells induced by one volunteer’s MoDCs. Statistical comparisons were also performed between young and elderly volunteers within each condition. Data shown as individual values, n = 8–10 young volunteers’ MoDCs, n = 8–10 elderly volunteers’ MoDCs, * = p<0.05, ** = p<0.005, *** = p<0.0005, **** = p<0.0001, comparing parent to daughter T cells.

Following interactions with young and elderly LPS/IFN-γ-MoDCs, increased percentages of daughter CD4^+^ T cells expressing CD25 (Fig 6A), and several regulatory markers (CD39, A2AR, IL-10 and TGF-β; Fig 6A), relative to parent CD4^+^ T cells were seen. Similarly, young and elderly LPS/IFN-γ-MoDCs induced increases in activation and regulatory markers on daughter compared to parent CD8^+^ T cells, specifically, increases in CD25, IL-12, CD39, A2AR, IL-10 and TGF-β (Fig 6B). These data imply that regardless of age, LPS/IFN-γ-MoDCs stimulate T cells with a similar phenotype, which in this case appears suppressive and may be accounted for by exposure duration (5 days).

In summary, elderly LPS/IFN-γ-MoDCs demonstrated comparable up-regulation of surface antigen-presenting and co-stimulatory molecules involved in T cell activation/priming as well as secretion of pro-inflammatory cytokines to their younger counterparts, suggesting that co-stimulation and activation of T cells is retained with aging. Elderly LPS/IFN-γ-MoDCs demonstrated an increased ability to stimulate CD8^+^ and CD4^+^ T cell T cell proliferation. However, MoDCs from elderly volunteers displayed impaired/partial responses to LPS/IFN-γ, shown by: (i) reduced ability to up-regulate MCP-1, IL-6 and IL-8; and (ii) partial down-regulation of antigen-processing capacity, suggesting incomplete MoDC maturation.

## Discussion

We examined whether the ability of DCs to respond to stimulation with CD40L or LPS/IFN-γ is altered during aging. We found that elderly and young-derived MoDCs generally responded equally to CD40L or LPS/IFN-γ activation. Although there were some differences, for example, elderly CD40L-activated MoDCs have increased capacity to present lipid antigens (due to increased CD1a), and may better activate effector T cells (due to increased CD40, CD80, CD86, and secreted IFN-γ and TNF-α). These observations suggest that elderly CD40L-activated DCs may activate effective anti-pathogen or anti-tumour T cell responses. However, our data also suggest that these T cells might not be activated sufficiently to proliferate extensively. Therefore, we also looked at the ability of elderly DCs to respond to LPS/IFN-γ, a milieu likely induced by a pathogen.

Following LPS/IFN-γ stimulation all elderly mDC1s, mDC2s, pDCs and MoDCs up-regulated similar levels of co-stimulatory molecules (CD40, CD80 and/or CD86) and intracellular pro-inflammatory cytokines (IFN-γ, TNF-α, IL-6 and/or IL-12), and secreted similar levels of IFN-α, IFN-γ and IL-12p70, to their younger counterparts. This suggests that in response to LPS/IFN-γ, elderly MoDCs remain robust in their ability to mature into functional APCs with the capacity to co-stimulate and activate T cells. Our findings agree with others showing that elderly human DCs up-regulate co-stimulatory molecules and pro-inflammatory cytokines in response to stimulation with LPS, influenza virus or influenza vaccines [31–39]. However, elderly individuals are reported to have increased susceptibility to viral infections and reduced vaccination responses [1]. Although our data shows that elderly DCs are relatively robust in their response to LPS/IFN-γ stimulation, we did not test their responsiveness to viruses or TLR-7/9 ligands, which other studies have shown are impaired [34, 40, 41]. Moreover, age-related defects in other immune cell compartments, particularly T and B cells, are also likely to contribute to the increased susceptibility of elderly individuals to viral infections, and reduced vaccination responses [1, 4]. Our observations show that elderly LPS/IFN-γ-MoDCs induced comparable, or greater, levels of CD8^+^ and CD4^+^ T cell proliferation, to their younger MoDC counterparts. This is similar to other studies showing that elderly DCs retain or increase their T cell stimulatory capacity [32, 33, 39, 42–46]. Importantly, elderly LPS/IFN-γ-MoDCs induced increased expression of CD25 and/or IL-12 on proliferating CD8^+^ and CD4^+^ T cells, suggesting that these T cells are activated and develop effector function. Furthermore, the ability of elderly MoDCs to respond to CD40L suggests that mature elderly DCs arriving at draining lymph nodes will be responsive to signals from CD40-CD40L interactions with CD4^+^ T cells, enabling licensing into potent APCs which activate T cells. Therefore, if mature LPS/IFN-γ-activated elderly DCs traffic to draining lymph nodes, they should provide appropriate positive co-stimulatory signals to prime/activate antigen-specific effector T cells.

Nonetheless, elderly-derived MoDCs did demonstrate a few differences in their responses to LPS/IFN-γ. For example, elderly MoDCs maintained their ability to process antigen. Down-regulation of antigen processing is typically considered a hallmark of DC maturation [10]. Therefore, maintenance of antigen processing suggests that elderly MoDCs may not fully mature in response to LPS/IFN-γ, yet they behaved as mature DCs and stimulated at least comparable levels of T cell proliferation, and induced similar T cell phenotypes, compared to young LPS/IFN-γ-MoDCs. It is possible that maintenance of antigen processing capacity by elderly LPS/IFN-γ mature DCs may be beneficial, as it may contribute to sustained processing of pathogen-derived antigens, leading to prolonged stimulation of pathogen-specific effector T cells. However, maintenance of antigen processing capacity by elderly mature DCs may be a double-edged sword, depending on the type of antigen processed. Down-regulation of antigen processing function may be a protective mechanism to limit processing self-antigens, and prevent generation of autoimmune responses [47–49]. If elderly mature DCs have sustained processing of self-antigens, this may lead to generation of self-reactive T cells, and autoimmunity [1, 21].

We also observed differences in chemokine/cytokine secretion by elderly LPS/IFN-γ-activated MoDCs. Elderly MoDCs did not consistently up-regulate the chemokines IL-8 and MCP-1 following stimulation with LPS/IFN-γ, suggesting that secretion of these chemokines may be impaired in a cohort of elderly individuals. IL-8 and MCP-1 are required for recruitment of neutrophils [50, 51] and monocytes [52], respectively, to a site of infection. Failure of elderly LPS/IFN-γ-MoDCs to secrete these chemokines may lead to reduced neutrophil and monocyte recruitment, a weakened inflammatory reaction at a site of infection, and impaired pathogen clearance. In contrast, other studies reported that elderly unstimulated DCs [34, 53] and DCs activated with a TLR-3 agonist [36] or influenza virus [34] secrete comparable levels of IL-8 or MCP-1 to their younger counterparts. It is possible that age-related defects in signalling via the TLR-4 [37] and/or IFN-γ pathways [54–56] in elderly DCs contribute to the impaired IL-8 and MCP-1 secretion we observed, explaining the contrasting results. However, heterogeneous responses in the elderly cohort may also contribute to the contrasting data. In addition, our data show that although elderly LPS/IFN-γ-MoDCs increased intracellular levels of IL-6, not all elderly MoDCs up-regulated secreted levels of IL-6. Others have also observed reduced IL-6 secretion by elderly DCs following stimulation with TLR agonists [41, 42, 53, 57, 58], including LPS [53, 57, 58]. IL-6 is required for differentiation and activation of Th2 and Th17 cells, which mediate responses against extracellular bacteria, fungi and parasites [59, 60]. Reduced IL-6 secretion by elderly DCs may lead to reduced generation of Th2 and Th17 cells, which could compromise immune responses against extracellular pathogens. Therefore, impairments in elderly DC chemokine/cytokine secretion may reduce the efficacy of elderly immune responses against infectious agents, and contribute to the increased susceptibility of elderly individuals to infections.

We observed that a greater number of elderly volunteers up-regulated latent TGF-β expression on their mDC1s, and PD-L1 expression on their pDCs following LPS/IFN-γ stimulation, relative to young volunteers. Additionally, when comparing each volunteer’s LPS/IFN-γ-stimulated sample to their own unstimulated control, the magnitude of increases in TGF-β on mDC1s and PD-L1 on pDCs was greater for elderly volunteers, compared to young volunteers. Together, this suggests that elderly mDC1s and pDCs may have greater potential to increase suppressive TGF-β and PD-L1 following LPS/IFN-γ stimulation. However, when comparing mDC1 TGF-β and pDC PD-L1 expression of young to elderly volunteers within unstimulated and LPS/IFN-γ-stimulated groups, no significant differences were observed implying that these potentially suppressive age-related differences may not be physiologically relevant. Further studies using a larger cohort of volunteers are required to confirm or refute age-related differences in TGF-β and PD-L1.

Future studies will extend our findings in a larger cohort and examine potential mechanisms underlying the changes we observed in elderly DCs. These include age-related changes in DC metabolism, signalling pathways and gene expression, and the effects of age-related changes in circulating factors known to influence DCs, such as cytokines, hormones and lipids. Additionally, we observed up-regulation of activation and regulatory markers on: (i) young and elderly DCs following stimulation with LPS/IFN-γ; and (ii) young T cells following interactions with young and elderly DCs, suggesting that at the time points we examined, DCs and T cells may be transitioning from the activation phase to the attenuation phase of an immune response. Therefore, analysis of DCs and T cells at earlier time points would be useful to determine whether there are age-related differences in their activation kinetics.

Strengths of this study include: (i) examination of a comprehensive range of DC phenotypic and functional attributes in response to LPS/IFN-γ and CD40L, to our knowledge, the effects of aging on DC responses to these stimuli have not yet been reported; (ii) examination of mDC1s, mDC2s and pDCs separately, whilst most other studies have grouped myeloid DC subsets together, and overlooked mDC2s; (iii) phenotypic analysis of elderly DCs included a range of regulatory markers, which have not been well-studied in the context of aging, and (iv) the use of fresh rather than stored frozen DCs to prevent storage/viability issues.

In summary, we have shown that elderly DCs mostly retain their capacity to respond to stimulation with LPS/IFN-γ or CD40L, as shown by similar magnitudes of changes in most phenotypic markers examined, relative to their younger counterparts. However, there were a few key differences which could impact elderly immune responses. Elderly MoDCs maintained their antigen processing ability following LPS/IFN-γ stimulation; this may allow sustained processing of pathogen-derived antigens and enhance anti-pathogen immune responses, however if self-antigen processing is sustained, this could contribute to increased autoimmunity. In addition, elderly LPS/IFN-γ-MoDCs may have (i) a reduced ability to recruit neutrophils and monocytes to a site of infection, due to reduced IL-8 and MCP-1; and (ii) a decreased ability to stimulate Th2 and Th17 responses, due to reduced IL-6 secretion. These compartmental defects in LPS/IFN-γ-activated elderly DCs may impact on the quality and effectiveness of anti-pathogen and anti-tumour immune responses, which could contribute to the increased susceptibility of elderly individuals to infection and cancer.

## Supporting information

S1 Fig Elderly CD40L-MoDCs have increased up-regulation of CD1a, CD40 and CD86. Young and elderly immature MoDCs were left unstimulated or stimulated with CD40L for two days, before flow cytometric analysis. CD11c^+^CD14^-^ MoDCs were identified within viable cells (A), large cells (B) and single cells (C) gates, and MoDCs positive for each marker measured; representative graph shown (E). Percentages of CD11c^+^CD14^-^ MoDCs positive for activation markers (F) were analysed. Concentrations of IFN-γ, TNF-α, IL-10, IL-12p70 and VEGF were measured in culture supernatants using a cytokine bead array (G). Each line in (F and G) represents an individual volunteer. Data shown as individual values, n = 10–14 young volunteers, n = 11–18 elderly volunteers, * = p<0.05, ** = p < 0.005, *** = p < 0.0005, **** = p < 0.0001 comparing (i) CD40L-MoDCs to unstimulated MoDCs from the same volunteer, or (ii) young to elderly CD40L-MoDCs.
(TIF)

S2 Fig Gating strategy for T cells from mixed lymphocyte reaction co-cultures. Young and elderly immature/unstimulated, LPS/IFN-γ-stimulated or CD40L-stimulated MoDCs were co-cultured with allogeneic, CFSE-labelled young T cells at DC: T cell ratios of 1:2, 1:5, 1:20, 1:50 and 1:200 for 5–8 days, then stained with CD3, CD4, and CD8 for flow cytometric analysis. Viable cells (A), single cells (B), then CD3^+^ T cells (C) were gated. Within the CD3^+^ gate, CD8^+^ and CD4^+^ T cells were identified (D). In each of the CD8^+^ and CD4^+^ T cell gates, parent and daughter T cells were identified based on CFSE staining intensity (E). The percentage of T cell proliferation (which corresponds to the daughter cells gate) was calculated based on loss of staining intensity of the parent peak (E).
(TIF)

S3 Fig Young and elderly mDC2s have similar responses to LPS/IFN-γ. Young and elderly PBMCs were left unstimulated or stimulated with LPS/IFN-γ for 24 hours, and analysed via flow cytometry for CD141^+^ mDC2s, and expression of activation (MHC-I, CD40, CD80, CD86, and intracellular TNF-α, IL-6 and IL-12) and regulatory markers (CD39, CD73, A2AR, A2BR, PD-L1, GAL-9, and intracellular IL-10 and TGF-β). Percentages of mDC2s positive for activation (A) and regulatory markers (B) were measured. Each line represents an individual volunteer, and compares their LPS/IFN-γ-stimulated sample to their unstimulated control. Statistical comparisons were also performed between young and elderly volunteers within each condition. Data shown as individual values, n = 10 young volunteers, n = 10 elderly volunteers, * = p<0.05, ** = p<0.005, *** = p<0.0005 comparing LPS/IFN-γ-mDC2s to unstimulated mDC2s from the same volunteer.
(TIF)

S4 Fig Young and elderly pDCs have similar responses to LPS/IFN-γ. Young and elderly PBMCs were left unstimulated or stimulated with LPS/IFN-γ for 24 hours, and analysed via flow cytometry for CD123^+^CD303^+^ pDCs, and expression of activation markers (MHC-I, CD40, CD80, CD86, and intracellular IFN-γ, TNF-α, IL-6 and IL-12), and regulatory markers (CD39, CD73, A2AR, A2BR, GAL-9, and intracellular IL-10 and TGF-β). Percentages of pDCs positive for activation (A) and regulatory markers (B) were measured. Each line represents an individual volunteer, and compares their LPS/IFN-γ-stimulated sample to their unstimulated control. Statistical comparisons were also performed between young and elderly volunteers within each condition. Data shown as individual values, n = 10 young volunteers, n = 10 elderly volunteers, * = p<0.05, ** = p<0.005 comparing LPS/IFN-γ-pDCs to unstimulated pDCs from the same volunteer.
(TIF)

S5 Fig Young and elderly MoDCs up-regulate IFN-α, IFN-γ, IL-12p70 and VEGF secretion in response to LPS/IFN-γ. Young and elderly monocytes were differentiated into immature MoDCs using GM-CSF and IL-4 for seven days, and left unstimulated or stimulated with LPS/IFN-γ for a further two days. Concentrations of IFN-α, IFN-γ, TNF-α, IL-1β, IL-10, IL-12p70, IL-17A, IL-18, IL-23, IL-33 and VEGF were measured in culture supernatants from young and elderly MoDCs via cytokine bead array (A and B); each line represents an individual volunteer, and compares their LPS/IFN-γ-stimulated sample to their unstimulated control. Statistical comparisons were also performed between young and elderly volunteers within each condition. Data shown as individual values, n = 10–22 young volunteers, n = 10–24 elderly volunteers, * = p<0.05, ** = p<0.005, *** = p<0.0005, **** = p<0.0001 comparing LPS/IFN-γ-MoDCs to unstimulated MoDCs from the same volunteer.
(TIF)
