# Supplementary figures and images for: Elderly dendritic cells respond to LPS/IFN-γ and CD40L stimulation despite incomplete maturation
Source: PLoS One. 2018 Apr 13;13(4):e0195313. doi: 10.1371/journal.pone.0195313 (PMC5898732; doi:10.1371/journal.pone.0195313)

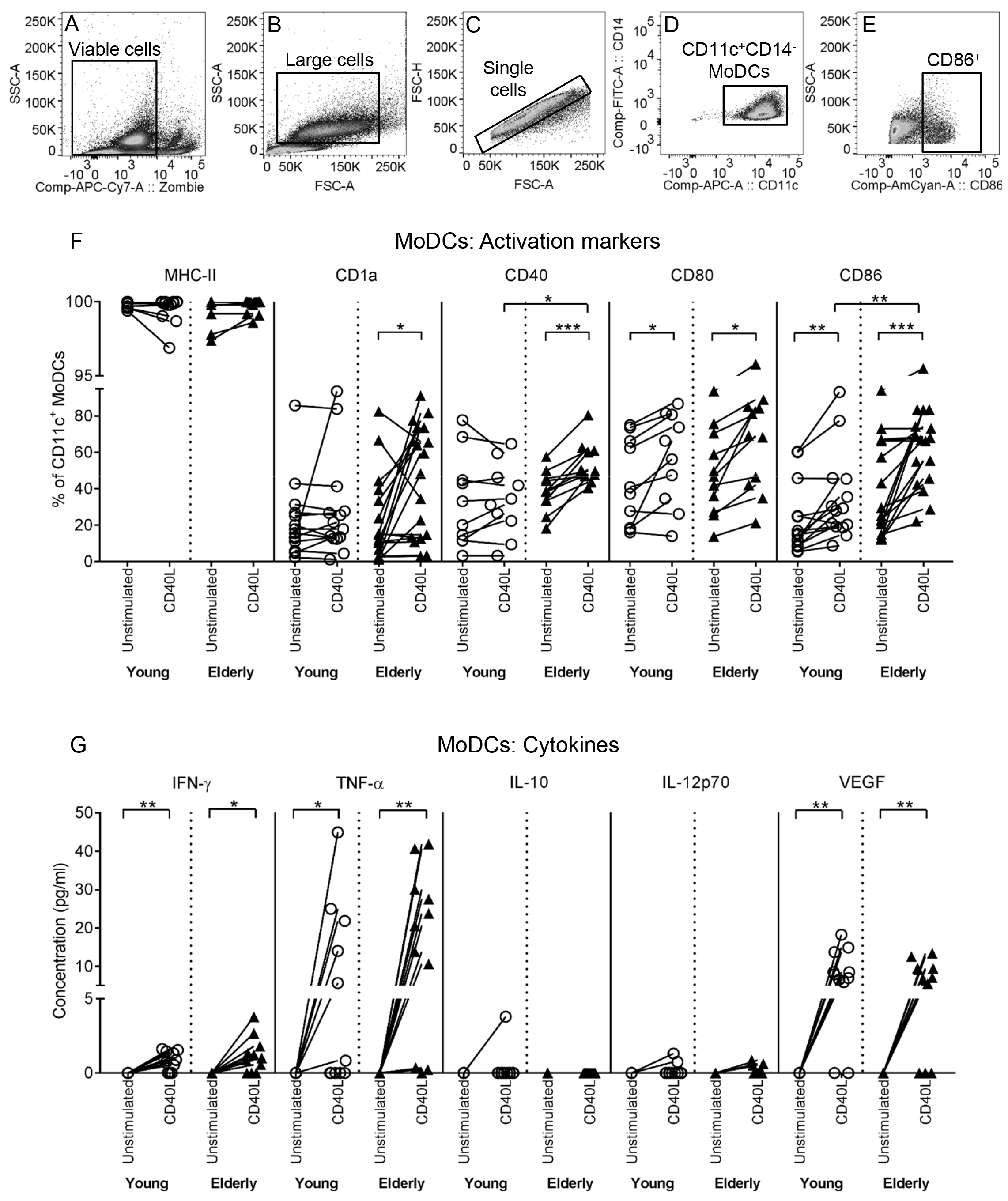

Supplement: S1 Fig — Young and elderly immature MoDCs were left unstimulated or stimulated with CD40L for two days, before flow cytometric analysis. CD11c+CD14- MoDCs were identified within viable cells (A), large cells (B) and single cells (C) gates, and MoDCs positive for each marker measured; representative graph shown (E). Percentages of CD11c+CD14- MoDCs positive for activation markers (F) were analysed. Concentrations of IFN-γ, TNF-α, IL-10, IL-12p70 and VEGF were measured in culture supernatants using a cytokine bead array (G). Each line in (F and G) represents an individual volunteer. Data shown as individual values, n = 10–14 young volunteers, n = 11–18 elderly volunteers, * = p<0.05, ** = p < 0.005, *** = p < 0.0005, **** = p < 0.0001 comparing (i) CD40L-MoDCs to unstimulated MoDCs from the same volunteer, or (ii) young to elderly CD40L-MoDCs. (TIF) [file pone.0195313.s001.tif]

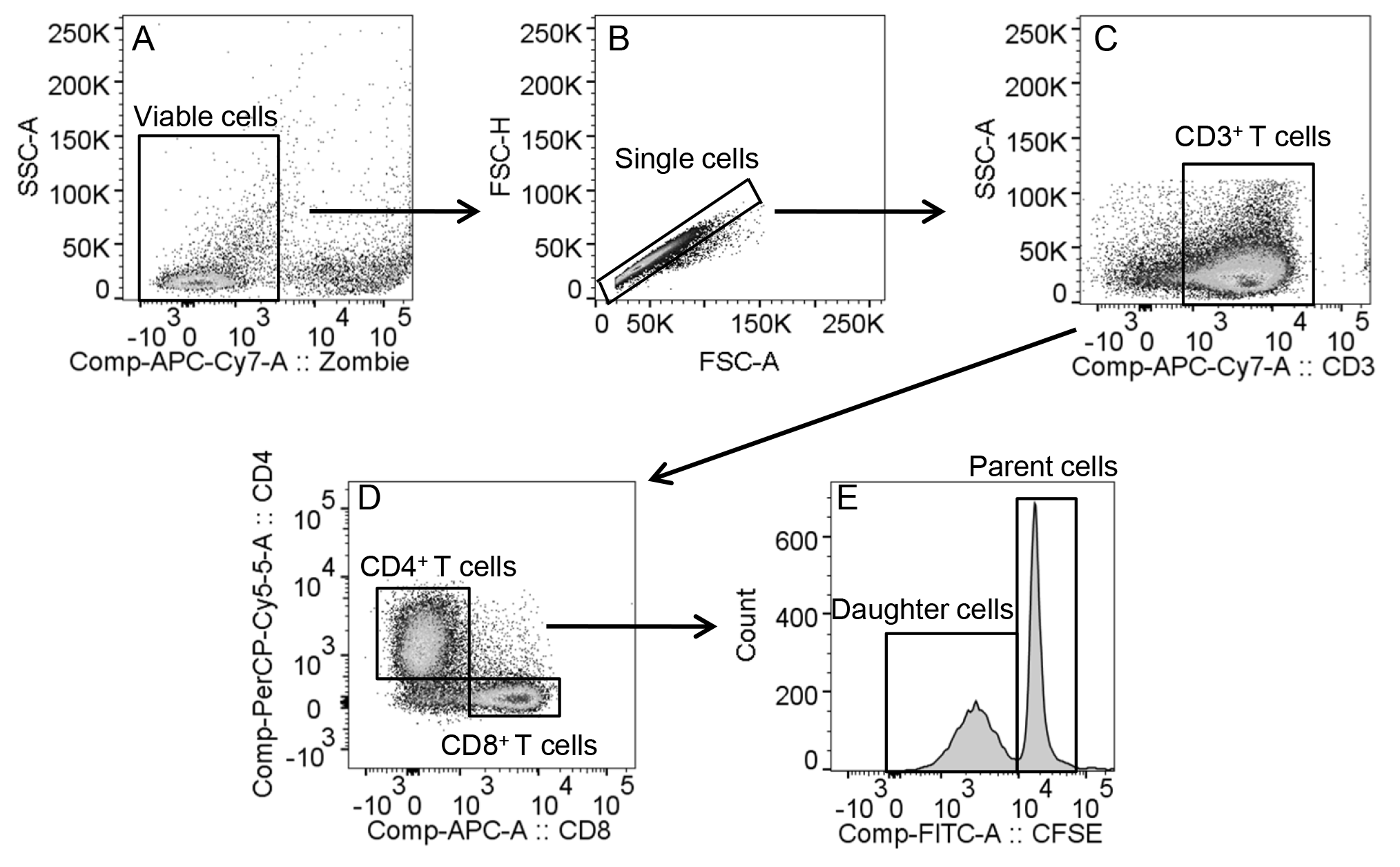

Supplement: S2 Fig — Young and elderly immature/unstimulated, LPS/IFN-γ-stimulated or CD40L-stimulated MoDCs were co-cultured with allogeneic, CFSE-labelled young T cells at DC: T cell ratios of 1:2, 1:5, 1:20, 1:50 and 1:200 for 5–8 days, then stained with CD3, CD4, and CD8 for flow cytometric analysis. Viable cells (A), single cells (B), then CD3+ T cells (C) were gated. Within the CD3+ gate, CD8+ and CD4+ T cells were identified (D). In each of the CD8+ and CD4+ T cell gates, parent and daughter T cells were identified based on CFSE staining intensity (E). The percentage of T cell proliferation (which corresponds to the daughter cells gate) was calculated based on loss of staining intensity of the parent peak (E). (TIF) [file pone.0195313.s002.tif]

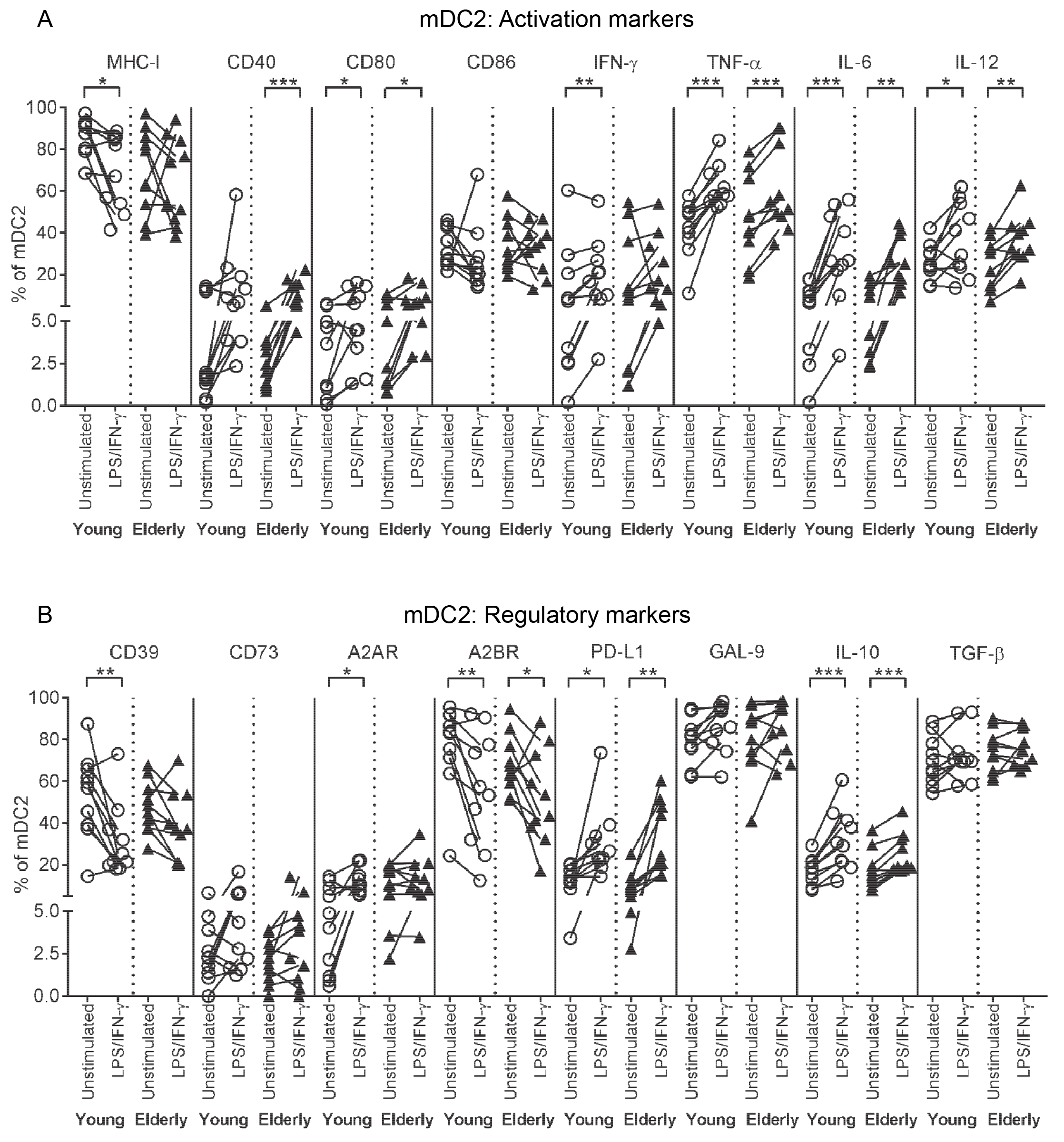

Supplement: S3 Fig — Young and elderly PBMCs were left unstimulated or stimulated with LPS/IFN-γ for 24 hours, and analysed via flow cytometry for CD141+ mDC2s, and expression of activation (MHC-I, CD40, CD80, CD86, and intracellular TNF-α, IL-6 and IL-12) and regulatory markers (CD39, CD73, A2AR, A2BR, PD-L1, GAL-9, and intracellular IL-10 and TGF-β). Percentages of mDC2s positive for activation (A) and regulatory markers (B) were measured. Each line represents an individual volunteer, and compares their LPS/IFN-γ-stimulated sample to their unstimulated control. Statistical comparisons were also performed between young and elderly volunteers within each condition. Data shown as individual values, n = 10 young volunteers, n = 10 elderly volunteers, * = p<0.05, ** = p<0.005, *** = p<0.0005 comparing LPS/IFN-γ-mDC2s to unstimulated mDC2s from the same volunteer. (TIF) [file pone.0195313.s003.tif]

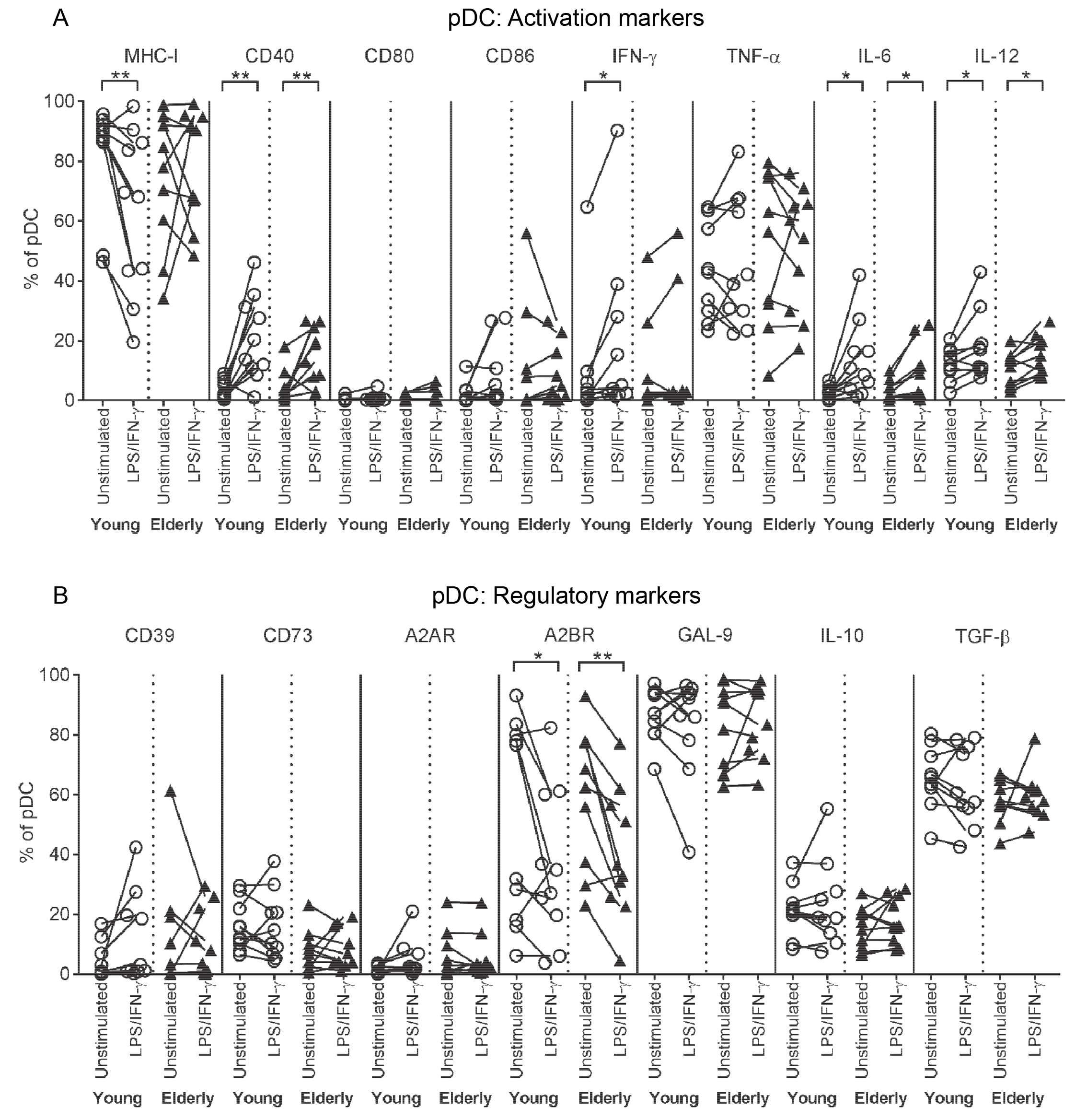

Supplement: S4 Fig — Young and elderly PBMCs were left unstimulated or stimulated with LPS/IFN-γ for 24 hours, and analysed via flow cytometry for CD123+CD303+ pDCs, and expression of activation markers (MHC-I, CD40, CD80, CD86, and intracellular IFN-γ, TNF-α, IL-6 and IL-12), and regulatory markers (CD39, CD73, A2AR, A2BR, GAL-9, and intracellular IL-10 and TGF-β). Percentages of pDCs positive for activation (A) and regulatory markers (B) were measured. Each line represents an individual volunteer, and compares their LPS/IFN-γ-stimulated sample to their unstimulated control. Statistical comparisons were also performed between young and elderly volunteers within each condition. Data shown as individual values, n = 10 young volunteers, n = 10 elderly volunteers, * = p<0.05, ** = p<0.005 comparing LPS/IFN-γ-pDCs to unstimulated pDCs from the same volunteer. (TIF) [file pone.0195313.s004.tif]

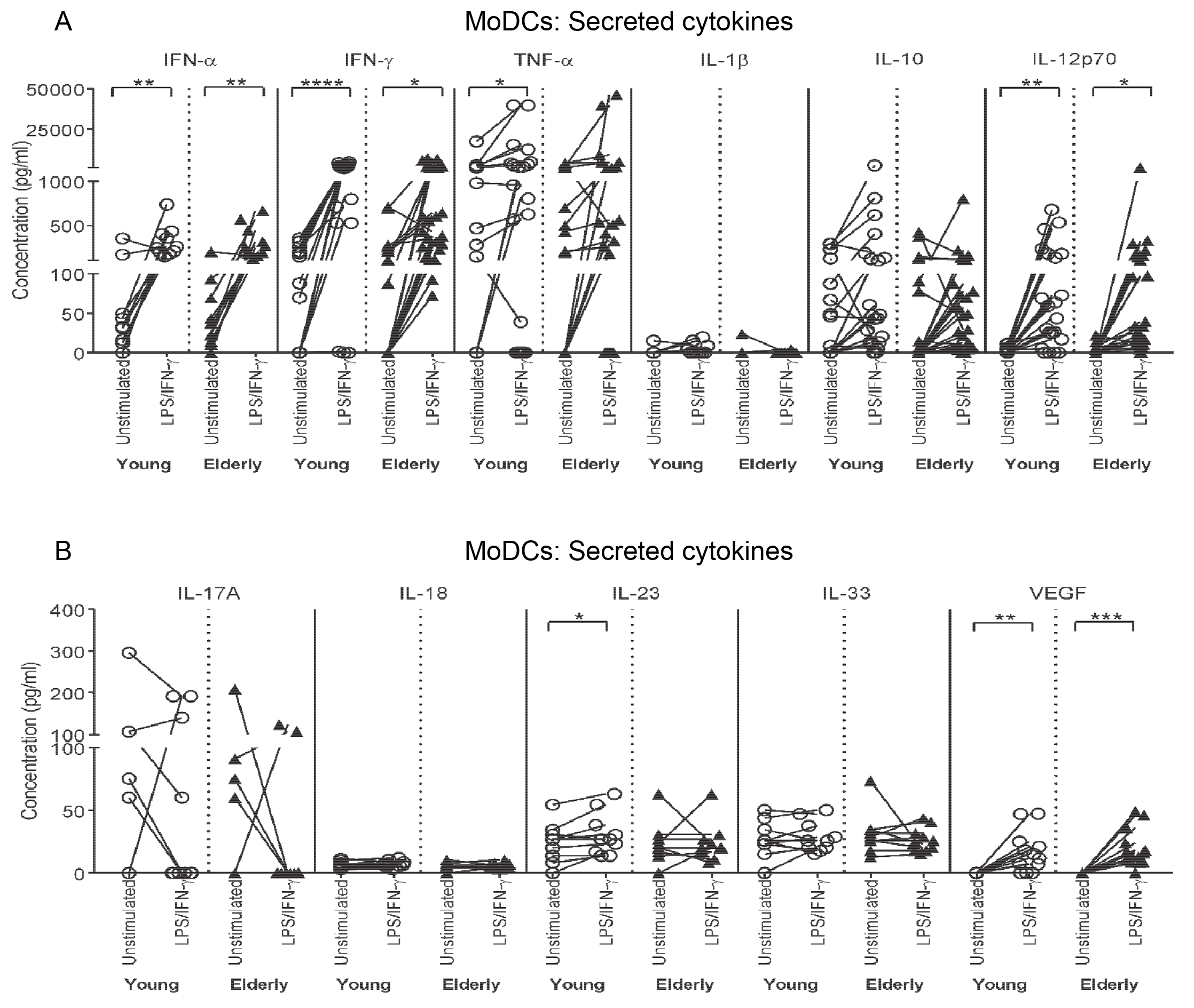

Supplement: S5 Fig — Young and elderly monocytes were differentiated into immature MoDCs using GM-CSF and IL-4 for seven days, and left unstimulated or stimulated with LPS/IFN-γ for a further two days. Concentrations of IFN-α, IFN-γ, TNF-α, IL-1β, IL-10, IL-12p70, IL-17A, IL-18, IL-23, IL-33 and VEGF were measured in culture supernatants from young and elderly MoDCs via cytokine bead array (A and B); each line represents an individual volunteer, and compares their LPS/IFN-γ-stimulated sample to their unstimulated control. Statistical comparisons were also performed between young and elderly volunteers within each condition. Data shown as individual values, n = 10–22 young volunteers, n = 10–24 elderly volunteers, * = p<0.05, ** = p<0.005, *** = p<0.0005, **** = p<0.0001 comparing LPS/IFN-γ-MoDCs to unstimulated MoDCs from the same volunteer. (TIF) [file pone.0195313.s005.tif]
